# Prostaglandin F2 and EP2 Agonists Exert Different Effects on 3D 3T3-L1 Spheroids during Their Culture Phase

**DOI:** 10.3390/biomedicines9121821

**Published:** 2021-12-02

**Authors:** Yosuke Ida, Masato Furuhashi, Megumi Watanabe, Araya Umetsu, Fumihito Hikage, Hiroshi Ohguro

**Affiliations:** 1 Departments of Ophthalmology, School of Medicine, Sapporo Medical University, S1W17, Chuo-ku, Sapporo 060-8556, Japan; funky.sonic@gmail.com (Y.I.); watanabe@sapmed.ac.jp (M.W.); araya.alaya.favreweissth@gmail.com (A.U.); fuhika@gmail.com (F.H.); 2 Departments of Cardiovascular, Renal and Metabolic Medicine, Sapporo Medical University, S1W17, Chuo-ku, Sapporo 060-8556, Japan; mfuruhas@gmail.com

**Keywords:** deepening of the upper eyelid sulcus (DUES), 3T3-L1 cells, PGF2α, EP2 agonists, Omidenepag, 3-dimensional (3D) tissue culture

## Abstract

To elucidate the effects of switching a PGF2α agonist, bimatoprost acid (BIM-A), to an EP2 agonist (Omidenepag—OMD; butaprost—Buta) or reversing the switching on adipose tissue, two-dimensional (2D) and three-dimensional (3D) cultures of 3T3-L1 cells were analyzed by lipid staining and according to the mRNA expression of adipogenesis-related genes (*Pparγ, Ap2*, and *Leptin*), components of the extracellular matrix (ECM; *collagen1* (*Col1*), *Col4*, *Col6*, and *fibronectin* (*Fn*)), and the sizes and stiffness of the 3D spheroids. Switching from BIM-A to EP2 agonists caused (1) suppression of lipid staining and downregulation of most adipogenesis-related genes, (2) smaller and stiffer 3D spheroids, and (3) upregulation of *Col1* and *Fn*, downregulation of *Col4* (2D), or up-regulation of all ECM genes (3D, BIM-A to OMD), as well as downregulation of *Col6* (3D, BIM-A to Buta). In contrast, reversing the switching resulted in (1) an enhancement in lipid staining (2D) and a significant upregulation of adipogenesis-related genes (2D, 3D Buta to BIM-A), (2) larger and slightly stiffer 3D spheroids, and (3) upregulation of *Col1* and *Fn* (2D). These collective findings indicate that the switching orders of BIM-A and EP2 agonists have a significant effect on lipid metabolism, ECM expression, and the physical stiffness of 3T3-L1 cells.

## 1. Introduction

Glaucomatous optic neuropathy (GON), a condition that is associated with elevated intraocular pressure (IOP), is a leading cause of irreversible blindness throughout the world [1,2], and the only effective evidenced-based therapies for GON are hypotensive therapies with medications and/or surgery [3]. Among these medications, prostaglandin F2α analogues (PGF2α-ags) are typically used as the first-line medication based upon their substantial hypotensive effects, as well as because they have fewer side effects [4]. Recently, however, as non-negligible periocular side effects, the deepening of the upper eyelid sulcus (DUES) and other types of prostaglandin-associated periorbitopathy (PAP) have been frequently reported among long-term users of PGF2α-ags [5]. Based on a magnetic resonance imaging study, orbital fat atrophy is primarily involved in the pathogenesis of DUES [6]. To support this evidence, an in vitro study using two-dimensional (2D) cultures of 3T3-L1 cells, which is the most common cell line used for lipid-related research, also confirmed the PGF2α-ags-induced suppression of adipogenesis [7]. In addition, to establish a more suitable in vivo model to replicate an adipocyte-spreading environment within a 3D conic space, a three-dimensional (3D) drop culture technique was used. In fact, this 3D in vivo model relatively accurately replicated a significant PGF2α-ags-induced modification in extracellular matrix (ECM) expression in addition to adipogenesis in 3T3-L1 cells [8], as well as in human orbital fibroblasts (HOFs) [9], and a difference in the frequency of occurrence of DUES among PGF2α-ags has been reported in several clinical studies [10,11,12,13].

As a new type of anti-glaucoma medication, a selective, non-prostaglandin, prostanoid EP2 agonist, Omidenepag isopropyl (OMDI), a prodrug that is hydrolyzed within the eye into the active form (Omidenepag—OMD) to express hypotensive effects, has now become available. Despite being a member of the same family of prostanoid receptor agonists, it was demonstrated that the effects of OMD on the pharmacokinetics of the aqueous humor (AH) were quite different from those of the commonly used PGF2α-ags [14]. These observations suggest that the EP2 agonist OMD may also cause different effects on orbital fatty tissues compared to those of PGF2α-ags. In fact, our pilot study using the 3D culture model with 3T3-L1 cells revealed that EP2 agonists induced significant enlargement of the 3D 3T3-L1 spheroid, although PGF2α-ags caused substantial down-sizing of the 3D 3T3-L1 spheroid [15]. Since it was revealed that both F2α and EP2 receptors are co-expressed with in the 3T3-L1 cells [15] and the effects on adipogenesis of the 3D 3T3-L1 spheroid were absolutely different between them, as above, it is of great interest whether or not both PGF2α-ags and EP2 agonist may affect each other in the adipogenesis of 3T3-L1 cells. From the perspective of clinical aspects, it would be of great interest to determine whether EP2 agonists compensate for the PGF2α-ags-induced suppression of adipogenesis of adipocytes and whether the first-line PGF2α-ags-induced atrophy of orbital fatty tissue could be avoided by switching to second-line EP2 agonists. Alternatively, from the perspective of non-clinical and basic scientific aspects, such switching of EP2 agonists and PGF2α-ags could reveal unidentified cross-linkages between these agonists and adipogenesis in 3T3-L1 cells.

Here, to study the interference effects of PGF2α-ags and EP2 agonists on adipogenesis of the 3D 3T3-L1 spheroid and the effects of switching from PGF2α-ag, bimatoprost-acid (BIM-A) to EP2 agonists—OMD and butaprost (Buta)—on adipogenesis, ECM expression, and the sizes and physical properties of the 3D spheroid, these results were compared with our previous results regarding mono-treatments with these drugs [8,15].

## 2. Materials and Methods

### 2.1. Two- or Three-Dimensional Cell Cultures of 3T3-L1 Cells under Conditions where Bimatoprost Acid (BIM-A) or EP2 Agonists Are Substituted for EP2 Agonists or BIM-A, Respectively

We used 3T3-L1 preadipocytes purchased from KAC (#EC86052701-G0, KAC, Kyoto, Japan), and the induction of adipogenic differentiation in two-dimensional (2D) or three-dimensional (3D) cultures was processed over 7 days, as described in a recent report [8]. Briefly, 2D-cultured 3T3-L1 cells in 2D growth medium (HG-DMEM containing 100 U/mL penicillin, 100 μg/mL streptomycin, and 10% CS) in a 150 mm dish at 37 °C were further processed to a 3D sphenoid culture in a 3D-grown medium (2D-grown medium supplemented with 0.25% *w/v* Methocel A4M). To obtain 3T3-L1 spheroids, approximately 20,000 cells in 28 μL of 3D spheroid medium were placed into each well of a drop culture plate (# HDP1385, Sigma-Aldrich, St. Louis, MO, USA, defined as 3D/Day 0). Thereafter, half of the medium (14 μL) was replaced daily with fresh medium in each well. For the induction of adipogenic differentiation, two days after the 2D-cultured cells in the 2D-grown medium or 3D spheroids at Day 1 in the 3D medium, they were supplemented with 250 nM dexamethasone, 10 nM T3, 10 μM troglitazone, and 1 μg/mL insulin during the initial two days, and over the following 4 days, they were supplemented with 10 μM troglitazone and 1 μg/mL insulin.

To study the effects of switching BIM-A to EP2 agonists, Omidenepag (OMD; a generous gift from Santen Pharmaceutical Co., Ltd., Osaka, Japan), butaprost (Buta; #13741, Funakoshi Co, Tokyo, Japan), or reverse-orders experiments were performed under the following 6 conditions: (1) preadipocytes of 3T3-L1 cells (DIF−); (2) their adipogenic differentiation (DIF+); (3) DIF+ with 100 nM BIM-A from Day 1 to Day 4, and thereafter, 100 nM BIM-A was replaced with 100 nM OMD for the following 3 days; (4) DIF+ with 100 nM OMD from Day 1 to Day 4, and thereafter, 100 nM OMD was replaced with 100 nM BIM-A for the following 3 days; (5) DIF+ with 100 nM BIM-A from Day 1 to Day 4, and thereafter, 100 nM BIM-A was replaced with 100 nM Buta for the following 3 days; (6) DIF+ with 100 nM Buta from Day 1 to Day 4, and thereafter, 100 nM Buta was replaced with 100 nM BIM-A for the following 3 days. The concentrations of the BIM-A and EP2 agonists used in the present study were determined to be optimal based on our previous study [15].

### 2.2. Lipid Staining of 2D- or 3D-Cultured 3T3-L1 Cells

Lipid staining of the 2D- and 3D-cultured 3T3-L1 cells, prepared as described above, was processed with an Oil Red O staining assay kit (Abcam, #133102) and BODIPY dye, respectively, as described previously [15,16]. Briefly, after washing with PBS, the 2D-cultured cells were fixed in a formalin solution for 15 min and stained with an Oil Red O solution for 30 min at room temperature (RT). Phase-contrast microscope images were taken to visualize the positive oil-stained droplets. For a quantitative analysis, the dye, which was extracted with isopropanol, was subjected to measurement of the O.D. at 500 nm. Alternatively, for BODIPY staining, 3D spheroids in 6 super-low-attachment well dishes were incubated in 0.2% BODIPY (# D3922, Thermo Fisher Scientific, Waltham, MA, USA) in PBS for 1 h and fixed in 4% paraformaldehyde (PFA) in PBS for 10 min at RT. Subsequently, they were incubated with Alexa Fluor 594 phalloidin (# 20553, Funakoshi) and DAPI (# D523, Dojindo) at 1:1000 dilutions for 3 h at RT. The fluorescence intensity of the BODIPY-stained lipid droplets was measured using a Nikon A1 confocal microscope (Tokyo, Japan) and quantified using Image J software version 2.0.0 (NIH, Bethesda, MD, USA).

### 2.3. Quantitative PCR and Micro-Indentation Force Analysis

Following reverse transcription and real-time PCR, total RNA extraction was processed as described previously [15,16]. The obtained cDNA levels are expressed as the fold change relative to those of the 36B4 (*Rplp0*) gene. Sequences of the primers and Taqman probes used are shown in Supplementary Table S1.

The micro-indentation force for the spheroids was measured with a micro-squeezer (CellScale, Waterloo, ON, Canada) as described previously [15,16]. Briefly, a single living spheroid was compressed between 3 × 3 mm plates to achieve a 50% deformation over a period of 20 s under monitoring with a microscopic camera. The required force (μN) was measured and the force/displacement (μN/μm) was calculated.

### 2.4. Statistical Analysis

All statistical analyses were performed using Graph Pad Prism 8 (GraphPad Software, San Diego, CA, USA) as recently described [8,15,16]. Data are demonstrated as the arithmetic mean ± the standard error of the mean (SEM).

## 3. Results

In our previous studies of the effects of the mono-treatment of PGF2α-ags and EP2 agonists using 2D- and 3D-cultured 3T3-L1 cells, we reported that both receptors are co-expressed within the 3T3-L1 cells, and that PGF2α-ags and EP2 agonists exert different effects on adipogenesis and the physical properties of the 2D- and 3D-cultured 3T3-L1 cells (summarized in Table 1) [8,15]. That is, PGF2α-ags induced downsizing and stiffer 3D 3T3-L1 spheroids, but the EP2 agonists had no effects on the physical properties of the spheroids, although both agonists significantly suppressed adipogenesis in the 2D- and 3D-cultured 3T3-L1 cells. In addition, we also showed that during the 7-day culture of the 3D spheroids, in the absence of drugs, (1) the downsizing and increased stiffness of the 3D spheroids progressed up to Day 5 and then reached a plateau, and (2) upon adipogenic differentiation (DIF+), such downsizing and the increase in stiffness were significantly inhibited, but differently. That is, the DIF+-induced effects on size and stiffness were evident earlier (by Days 3 to 5) and later (during Days 5 to 7), respectively [16]. These collective findings indicate that the effects of these agonists on size and stiffness reflect those of the earlier and later phases of the physical properties of the 3D spheroids. Therefore, to elucidate the interference effects of BIM-A, which induces the most prominent effect on the occurrence of DUES [10,17] among the PGs, and EP2 agonists—OMD and Buta—on the earlier and later phases of the physical properties of the 3D spheroids, we evaluated several combinations in which the order of administration of the BIM-A and EP2 agonists (OMD or Buta) on adipogenesis and ECM expression in 2D- and 3D-cultured 3T3-L1 cells was switched, in addition to measuring the physical stiffness of the resulting 3D spheroids.

### 3.1. Effects of Switching the BIM-A and EP2 Agonists (OMD or Buta) on Adipogenesis and ECM Expression in 2D-Cultured 3T3-L1 Cells

**Figure 1:**
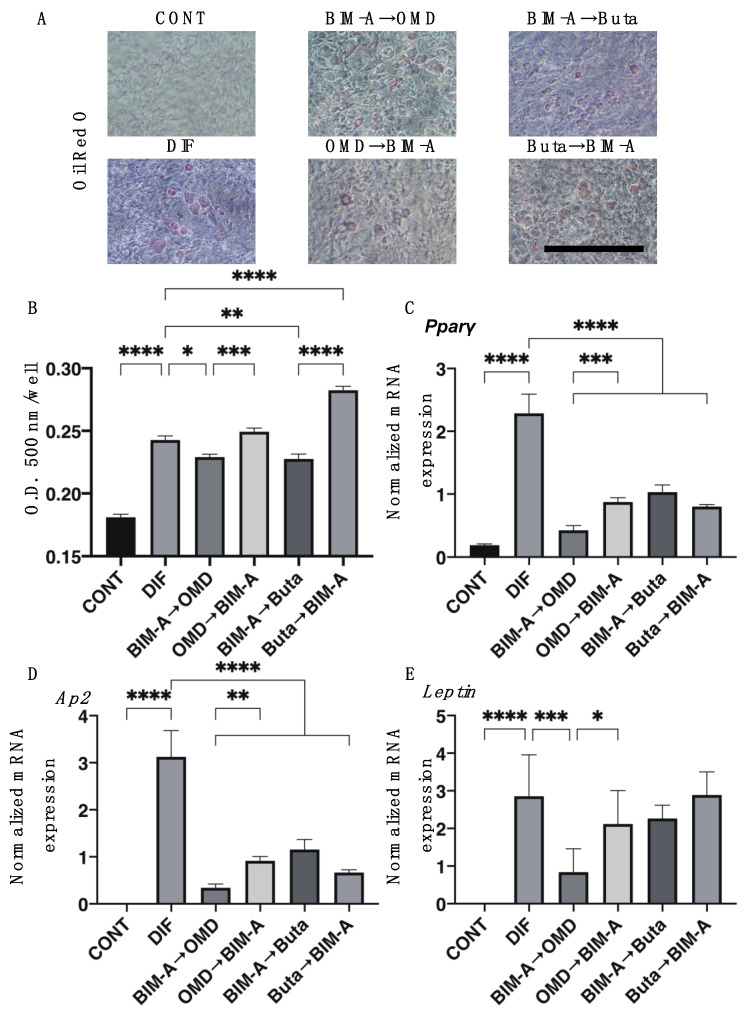
Effects of switching from bimatoprost acid (BIM-A) to EP2 agonists or from EP2 agonists to BIM-A on adipogenesis of 2D-cultured 3T3-L1 cells. The 3T3-L1 cells were 2D cultured under the following six conditions; (1) preadipocytes of 3T3-L1 cells (DIF−); (2) their adipogenic differentiation (DIF+); (3) DIF+ with 100 nM BIM-A from Day 1 to Day 4, and thereafter, 100 nM BIM-A was replaced with 100 nM Omidenepag (OMD) for the following 3 days; (4) DIF+ with 100 nM OMD from Day 1 to Day 4, and thereafter, 100 nM OMD was replaced with 100 nM BIM-A for the following 3 days; (5) DIF+ with 100 nM BIM-A from Day 1 to Day 4, and thereafter, 100 nM BIM-A was replaced with 100 nM butaprost (Buta) for the following 3 days; (6) DIF+ with 100 nM Buta from Day 1 to Day 4, and thereafter, 100 nM Buta was replaced with 100 nM BIM-A for the following 3 days. These specimens were subjected to analysis by Oil Red O lipid staining (panel **A**: representative phase-contrast images, scale bar: 100 μm; panel (**B**): their staining intensities, O.D.) and qPCR of *Pparγ*, *Ap2,* and *Leptin* (panels **C**–**E**). All experiments were performed in triplicate using fresh preparations (n = 5 each for lipid staining and qPCR analysis for one analysis). Data are presented as the arithmetic mean ± the standard error of the mean (SEM). * *p* < 0.05, ** *p* < 0.01, *** *p* < 0.005, **** *p* < 0.001 (ANOVA followed by Tukey’s multiple-comparison test).

**Figure 2:**
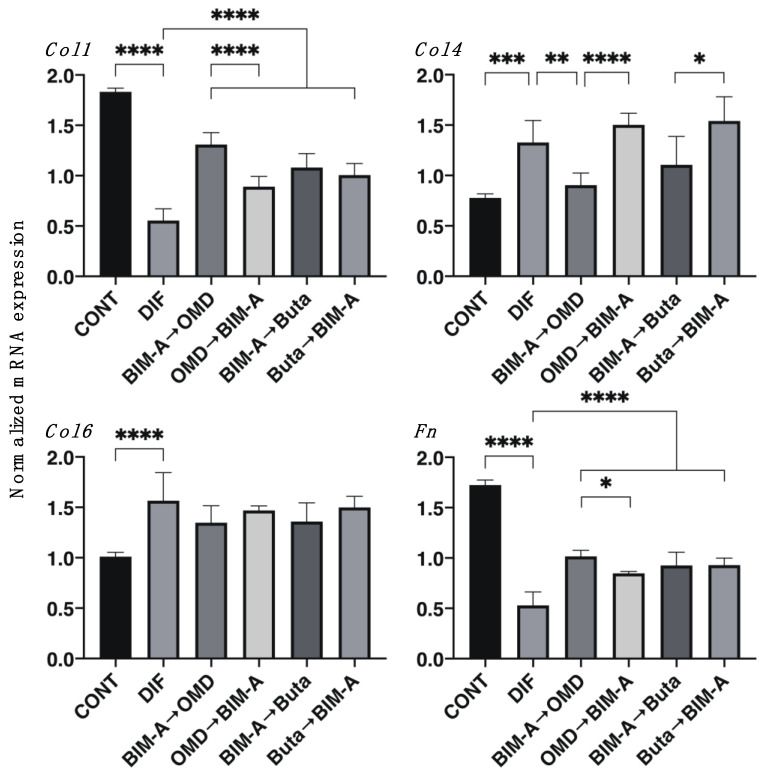
Effects of switching bimatoprost acid (BIM-A) to EP2 agonists or EP2 agonists to BIM-A on the mRNA expression of ECMs of 2D-cultured 3T3-L1 cells. The 3T3-L1 cells were 2D cultured under the following six conditions: (1) preadipocytes of 3T3-L1 cells (DIF−); (2) their adipogenic differentiation (DIF+); (3) DIF+ with 100 nM BIM-A from Day 1 to Day 4, and thereafter, 100 nM BIM-A was replaced with 100 nM Omidenepag (OMD) for the following 3 days; (4) DIF+ with 100 nM OMD from Day 1 to Day 4, and thereafter, 100 nM OMD was replaced with 100 nM BIM-A for the following 3 days; (5) DIF+ with 100 nM BIM-A from Day 1 to Day 4, and thereafter, 100 nM BIM-A was replaced with 100 nM butaprost (Buta) for the following 3 days; (6) DIF+ with 100 nM Buta from Day 1 to Day 4, and thereafter, 100 nM Buta was replaced with 100 nM BIM-A for the following 3 days. These specimens were subjected to a qPCR analysis to estimate the mRNA expression of the major ECM proteins (*Col1*: collagen 1, *Col4*: collagen 4, *Col6*: collagen 6, *Fn*: fibronectin). All experiments were performed in triplicate using fresh preparations (n = 5 each for one analysis). Data are presented as the arithmetic mean ± the standard error of the mean (SEM). * *p* < 0.05, ** *p* < 0.01, *** *p* < 0.005, **** *p* < 0.001 (ANOVA followed by Tukey’s multiple-comparison test).

As shown in Figure 1, the lipid staining with Oil Red O and the quantitative PCR of adipogenesis-related genes, including *Pparγ*, *Ap2,* and *Leptin,* of the 2D-cultured 3T3-L1 cells showed a significant enhancement upon adipogenesis (DIF+), which is consistent with our previous studies [8,15]. In comparison to DIF+, the Oil Red O staining intensities were significantly decreased upon switching from BIM-A to EP2 agonists (OMD or Buta), while a significant enhancement in lipid staining intensities was observed in the case of the switching of Buta to BIM-A (Figure 1, panel B). As for the mRNA expression of adipogenesis-related genes, including *Pparγ*, *Ap2,* and *Leptin*, the switching of BIM-A to OMD caused significant downregulation of all three genes of DIF+. In contrast, in other switching combinations, the DIF+-induced mRNA expression of *Pparγ* and *Ap2* was also downregulated but those effects were less than those when switching BIM-A to OMD, and those of *Leptin* were not significantly altered. Consistently with previous studies [8,15], the mRNA expression of *Col1* and *Fn* or *Col4* and *Col6* was downregulated or upregulated, respectively, by DIF+ (Figure 2). By switching from BIM-A to the EP2 agonists or the reverse order, *Col1* and *Fn* expression was significantly upregulated, but the expression of *Col6* was not altered as compared to that with DIF+. In terms of *Col4* expression, it was downregulated upon switching from BIM-A to the EP2 agonists.

### 3.2. Effects of Switching BIM-A and EP2 Agonists (OMD or Buta) on Physical Properties, Size, Stiffness, Adipogenesis, and ECM Expression in 3D 3T3-L1 Spheroids

**Figure 3:**
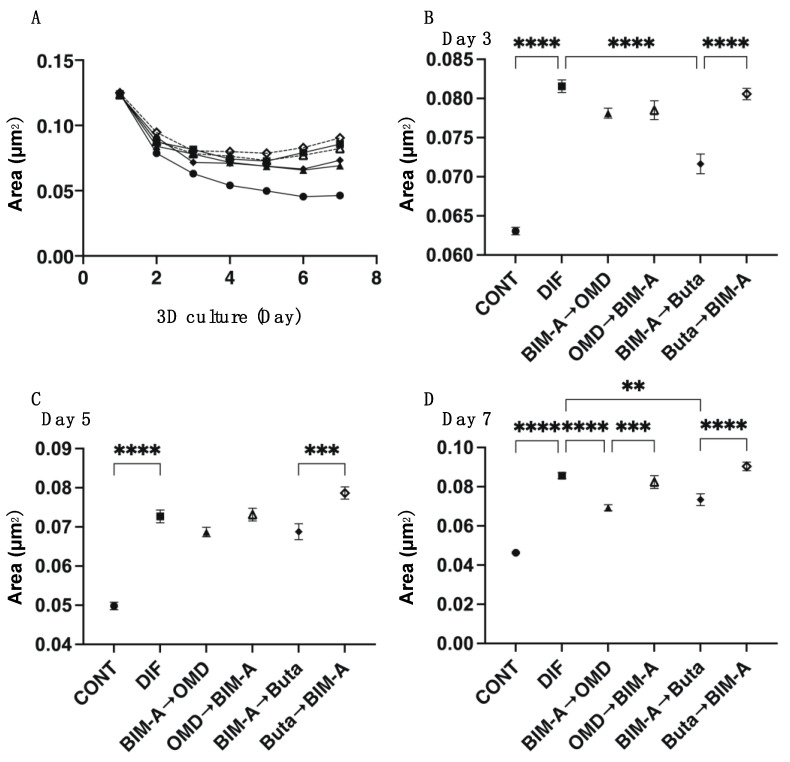
Effects of switching bimatoprost acid (BIM-A) to EP2 agonists or EP2 agonists to BIM-A on the mean area sizes of 3D 3T3-L1 spheroids during adipogenesis. The 3D 3T3-L1 spheroid cell cultures were processed under the following six conditions: (1) preadipocytes of 3T3-L1 cells (DIF−); (2) their adipogenic differentiation (DIF+); (3) DIF+ with 100 nM BIM-A from Day 1 to Day 4, and thereafter, 100 nM BIM-A was replaced with 100 nM Omidenepag (OMD) for the following 3 days; (4) DIF+ with 100 nM OMD from Day 1 to Day 4, and thereafter, 100 nM OMD was replaced with 100 nM BIM-A for the following 3 days; (5) DIF+ with 100 nM BIM-A from Day 1 to Day 4, and thereafter, 100 nM BIM-A was replaced with 100 nM butaprost (Buta) for the following 3 days; (6) DIF+ with 100 nM Buta from Day 1 to Day 4, and thereafter, 100 nM Buta was replaced with 100 nM BIM-A for the following 3 days. Their mean area sizes (μm^2^) were measured and plotted over a 7-day culture period (panel **A**). Those at Day 3 (panel **B**), Day 5 (panel **C**), and Day 7 (panel **D**) were compared among the experimental groups. All experiments were performed in triplicate using fresh preparations (n = 16 each for one analysis). Data are presented as the arithmetic mean ± the standard error of the mean (SEM). ** *p* < 0.01, *** *p* < 0.005, **** *p* < 0.001 (ANOVA followed by Tukey’s multiple-comparison test).

**Figure 4:**
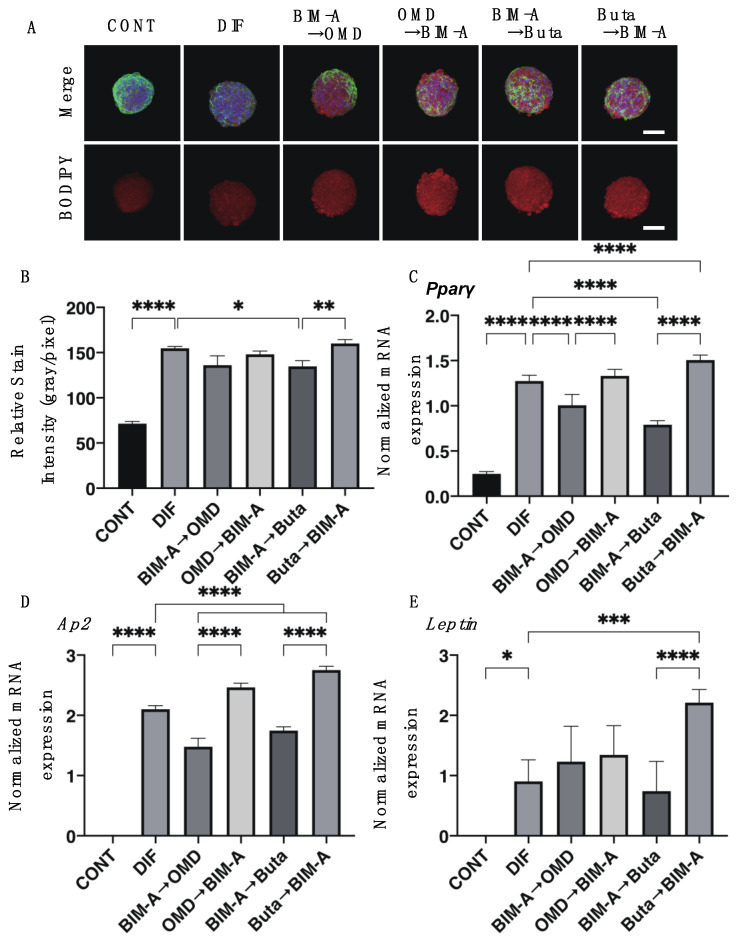
Effects of switching bimatoprost acid (BIM-A) to EP2 agonists or EP2 agonists to BIM-A on adipogenesis of 3D 3T3-L1 spheroids. The 3D 3T3-L1 spheroid cell cultures were processed under the following six conditions: (1) preadipocytes of 3T3-L1 cells (DIF−); (2) their adipogenic differentiation (DIF+); (3) DIF+ with 100 nM BIM-A from Day 1 to Day 4, and thereafter, 100 nM BIM-A was replaced with 100 nM Omidenepag (OMD) for the following 3 days; (4) DIF+ with 100 nM OMD from Day 1 to Day 4, and thereafter, 100 nM OMD was replaced with 100 nM BIM-A for the following 3 days; (5) DIF+ with 100 nM BIM-A from Day 1 to Day 4, and thereafter, 100 nM BIM-A was replaced with 100 nM butaprost (Buta) for the following 3 days; (6) DIF+ with 100 nM Buta from Day 1 to Day 4, and thereafter, 100 nM Buta was replaced with 100 nM BIM-A for the following 3 days. These samples were immunostained with DAPI (blue), phalloidin (green), and BODIPY (red). Merged images and BODIPY images are shown in panel **A** (scale bar: 100 μm), and their staining intensities (gray/pixel) were plotted (panel **B**). The mRNA expression of adipogenesis-related genes, including *Pparγ*, *Ap2,* and *Leptin,* under the above conditions was plotted, and the data are shown in panels (**C**–**E**). All experiments were performed in duplicate using fresh preparations (n = 5 each for BODIPY staining and 16 each for qPCR analysis for one analysis). Data are presented as the arithmetic mean ± the standard error of the mean (SEM). * *p* < 0.05, ** *p* < 0.01, *** *p* < 0.005, **** *p* < 0.001 (ANOVA followed by Tukey’s multiple-comparison test).

**Figure 5:**
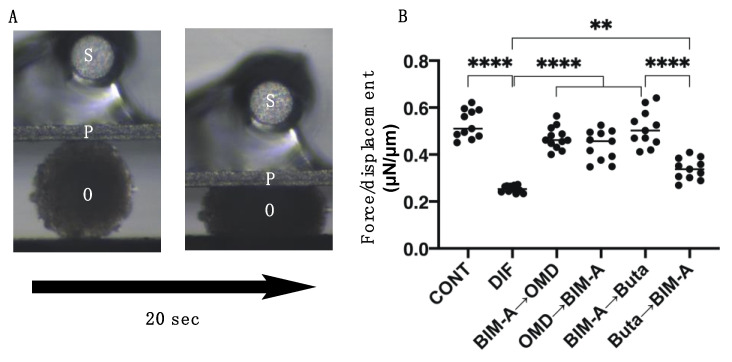
Effects of switching bimatoprost acid (BIM-A) to EP2 agonists or EP2 agonists to BIM-A on the physical stiffness of 3D 3T3-L1 spheroids. The 3D 3T3-L1 spheroid cell cultures were processed under the following six conditions: (1) preadipocytes of 3T3-L1 cells (DIF−); (2) their adipogenic differentiation (DIF+); (3) DIF+ with 100 nM BIM-A from Day 1 to Day 4, and thereafter, 100 nM BIM-A was replaced with 100 nM Omidenepag (OMD) for the following 3 days; (4) DIF+ with 100 nM OMD from Day 1 to Day 4, and thereafter, 100 nM OMD was replaced with 100 nM BIM-A for the following 3 days; (5) DIF+ with 100 nM BIM-A from Day 1 to Day 4, and thereafter, 100 nM BIM-A was replaced with 100 nM butaprost (Buta) for the following 3 days; (6) DIF+ with 100 nM Buta from Day 1 to Day 4, and thereafter, 100 nM Buta was replaced with 100 nM BIM-A for the following 3 days. The specimens were collected on Day 7 and were subjected to a physical solidity analysis. A single 3D spheroid was placed on a 3 × 3 mm plate and was then compressed to 50% deformation over a period of 20 s while being continuously monitored with a microscopic camera (panel **A**); S: micro-sensor of the mechanical force (μN), P: compression plate, O: single 3D spheroid). Under the experimental conditions listed above, the required force (μN) was measured and the force/displacement (μN/μm) was plotted (panel **B**) (n = 10 each). ** *p* < 0.01, **** *p* < 0.001 (ANOVA followed by Tukey’s multiple-comparison test).

We next examined the effects of switching the BIM-A and EP2 agonists on 3D 3T3-L1 spheroids, which our group recently established as a suitable in vivo model for DUES [8,15]. As shown in Figure 3, consistently with our previous study, (1) the sizes of the DIF− 3D 3T3-L1 spheroids became smaller during the 7-day culture, (2) upon DIF+, their mean area sizes increased significantly, and (3) these DIF+-induced effects were significantly inhibited on the switching of BIM-A to EP2 agonists, but not in the reverse order. Since, as stated above, the changes in the 3D spheroids were almost complete by Day 5 and the effects of the mono-treatment of BIM-A but not of the EP2 agonists caused downsizing effects, these results were quite understandable. Similar effects of switching were also observed in their lipid staining with BODIPY and the expression of the adipogenesis genes of *Pparγ* and *Ap2*. However, in contrast, the expression of *Leptin* was significantly upregulated in the case of switching from Buta to BIM-A (Figure 4). Physical stiffness analyses indicated that the 3D 3T3-L1 spheroids became less stiff upon DIF+ (Figure 5). These DIF+-induced effects were significantly reduced by the action of the BIM-A and EP2 agonists, although these reducing efficacies also greatly fluctuated with their switching orders. In terms of adipogenesis and the physical stiffness of the 3D spheroids, since we showed that these changes were more evident in the later (Days 5 to 7) rather than the earlier (until Day 5) period during the course of the 3D cultures [8,16], the unexpected results for *Pparγ* in the expression of *Ap2* observed in cases of switching from OMD or Buta to BIM-A, which was assumed to be due to the effects of a mono-treatment, may have been caused by some unknown cross-linkages between EP2 and PGF2α-ags receptors that are related to signaling (Table 1).

**Figure 6:**
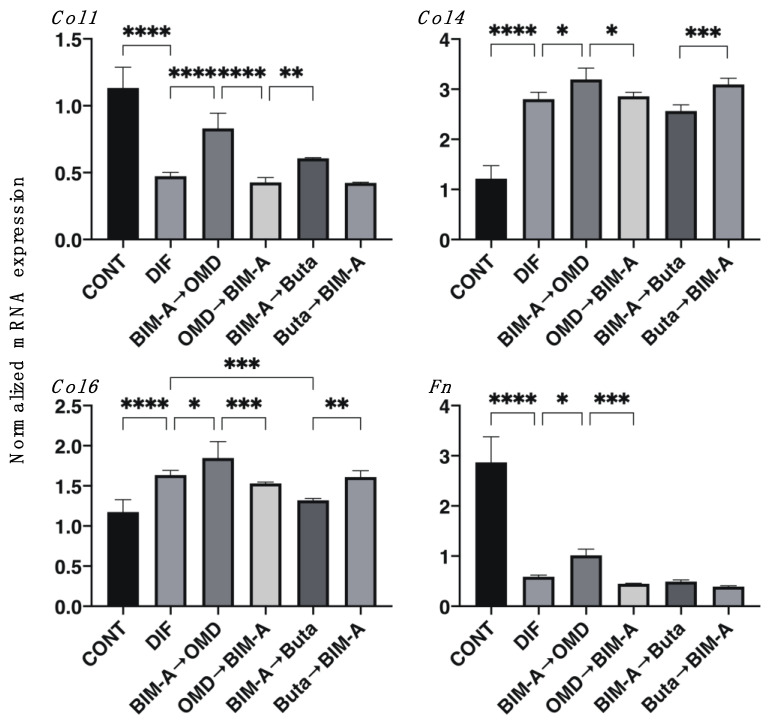
Effects of switching bimatoprost acid (BIM-A) to EP2 agonists or EP2 agonists to BIM-A on the mRNA expression of ECMs in 3D 3T3-L1 spheroids. The 3D 3T3-L1 spheroid cell cultures were processed under the following six conditions: (1) preadipocytes of 3T3-L1 cells (DIF−); (2) their adipogenic differentiation (DIF+); (3) DIF+ with 100 nM BIM-A from Day 1 to Day 4, and thereafter, 100 nM BIM-A was replaced with 100 nM Omidenepag (OMD) for the following 3 days; (4) DIF+ with 100 nM OMD from Day 1 to Day 4, and thereafter, 100 nM OMD was replaced with 100 nM BIM-A for the following 3 days; (5) DIF+ with 100 nM BIM-A from Day 1 to Day 4, and thereafter, 100 nM BIM-A was replaced with 100 nM butaprost (Buta) for the following 3 days; (6) DIF+ with 100 nM Buta from Day 1 to Day 4, and thereafter, 100 nM Buta was replaced with 100 nM BIM-A for the following 3 days. The specimens collected on Day 7 were subjected to a qPCR analysis to estimate the mRNA expression of ECM components (*Col1*: collagen 1, *Col4*: collagen 4, *Col6*: collagen 6, *Fn*: fibronectin). All experiments were performed in duplicate using fresh preparations (n = 16 each for one analysis). Data are presented as the arithmetic mean ± the standard error of the mean (SEM). * *p* < 0.05, ** *p* < 0.01, *** *p* < 0.005, **** *p* < 0.001 (ANOVA followed by Tukey’s multiple-comparison test).

To study this further, the effects caused by the switching of BIM-A and EP2 agonists on the mRNA expression of major ECM components, including *Col1, Fn*, *Col4,* and *Col6,* were determined. As shown in Figure 6, DIF+ induced a significant upregulation of *Col4* and *Col6,* and the downregulation of *Col1* and *Fn* was observed, similarly to the 2D-cultured experiment described above. DIF+-induced ECM expression was not significantly altered, except that all of the ECMs were upregulated in the case of switching BIM-A to OMD. Therefore, these collective data indicate that switching the order of BIM-A and EP2 agonists significantly affected the physical properties of the 3D 3T3-L1 spheroids, i.e., their size and stiffness, as well as the efficacies of their adipogenesis, despite having fewer effects on ECM expression.

## 4. Discussion

The types of PGE2 linked to four G-protein-coupled receptor subtypes are referred to as prostaglandin receptors EP1 through EP4 [18]. EP1 stimulation induces an influx of calcium to enhance intracellular free calcium levels [19]. Alternatively, EP2 or EP4 stimulation increases cAMP levels, whereas EP3 stimulation inhibits cAMP production. In addition to this, these EP species also exert their action via different regulatory mechanisms and signal transduction pathways. Among these, several in vivo studies have reported that the EP2 receptor is involved in IOP regulation and EP2 receptor agonists cause ocular hypotensive effects on elevated IOPs [14]. OMDI, a non-prostanoid isopropyl ester derivative, is hydrolyzed to OMD during its corneal penetration, with little or no activity for EP4 receptors [20]. OMDI was reported to have strong effects on lowering IOP in various animal models of OH and glaucoma [14], as well as in patients with GON and OH, with efficacies for lowering IOP comparable to those of latanoprost (LAT) [21]. In terms of the effects on adipogenesis, a number of studies have reported that PGE2 and PGF2α both inhibit adipocyte development [22,23,24].

It is generally thought that adipogenic differentiation from precursor stem cells into adipocytes involves two steps—namely, determination and terminal differentiation—and these processes are critically regulated by regulator genes of adipogenesis, including PPARγ, and the CCAAT enhancer-binding protein α (C/EBPα), as well as other metabolic genes, including Ap2 and Leptin [25]. In terms of the effects of EP2 agonists on adipogenesis, ONO-AE1-259, a selective EP2 receptor agonist, caused improvement of ectopic fat accumulation and glucose homeostasis with restoration of the capacity for storage of excess energy in subcutaneous adipose tissue based on ONO-AE1-259-induced activation of adipose angiogenesis and adipogenesis in a murine model of elastase-induced pulmonary emphysema (EIE mice) [26]. Based on this, it was suggested that EP2 agonists may become a therapeutic option as pro-angiogenic agents targeting the vascular component of adipose tissue for weight loss in patients with chronic obstructive pulmonary disease (COPD), and particularly in those with severe emphysema [27]. In terms of the effects of the EP2 agonist OMD on adipogenesis, we recently found that OMD caused significant suppression of the adipogenesis of 2D- and 3D-cultured 3T3-L1 cells [15], which is consistent with a number of studies demonstrating PGE2-dependent inhibition of adipocyte development [22,23].

It was revealed that EP2 receptors are often co-expressed with FP receptors in the same cells, and both function differently within intracellular signaling pathways; that is, EP2 receptors couple to Gαs and increase cAMP, and FP receptors couple to Gαq and release inositol-1,4,5-triphosphate (IP) [28,29] and dialcylglycerol (DAG) [28,30,31]. Similarly, such co-expression of EP2 and PF receptors was also recognized in 3T3-L1 preadipocytes, and we recently found that the effects of the EP2 agonist OMD on 2D- and 3D-cultured 3T3-L1 cells were different from those of PGF2α [15]. Therefore, if PGF2α-ags or EP2 agonists exert different effects on these complex mechanisms during the adipogenesis, as shown above, it would be of great interest to investigate the effects in the case of switching from PGF2α-ags to EP2 agonists—or the reverse—during the adipogenesis of 3T3-L1 cells. The findings reported herein provide the following observations: (A) switching from BIM-A to EP2 agonists induced (1) significantly downsized and stiffened 3D 3T3-L1 spheroids, (2) suppression of lipid staining (2D and 3D) and the downregulation of adipogenesis-related genes (2D and 3D, except for *Leptin* expression), and (3) the upregulation of the expression of *Col1* and *Fn* and downregulation of *Col4* (2D), while, in contrast, all four ECM components were upregulated in the case of switching from BIM-A to OMD (3D), and (B) switching the EP2 agonists to BIM-A resulted in (1) stiffened 3D 3T3-L1 spheroids, (2) enhanced lipid staining (2D), and a significant downregulation of the expression of *Pparγ* and *Ap2* (2D) or the upregulation of adipogenesis-related genes (3D). These data indicate that switching the order of BIM-A and EP2 agonists greatly affected adipogenesis in 2D- and 3D-cultured 3T3-L1 cells, as opposed to their ECM expression. Therefore, these collective findings demonstrated that switching from BIM-A to EP2 agonists inhibited the lipid staining and mRNA expression of adipogenesis-related genes in both 2D- and 3D-cultured 3T3-L1 cells. However, interestingly, the reverse switching caused significantly different effects, that is, lipid staining, as well as the mRNA expression of adipogenesis-related genes, was enhanced. Similarly, these switching orders also greatly affected the mRNA expression of in the ECMs and the physical stiffness of the 3D 3T3-L1 spheroids. As of the writing of this text, the issue of why these differences are caused by the order of switching remain to be elucidated. However, we speculate that this may be caused by the timing of the treatments with the drugs because of the differences in the natures of the 3D 3T3-L1 spheroids during the course of their culture, that is, early stage (until Day 5) vs. late stage (Days 5 to 7) [8,16], as well as some yet-to-be-identified cross-linkages between EP2 and PGF2α-ags that mediate signaling, as discussed above (Table 1). Furthermore, from the clinical point of view, these observations suggest that if an EP2 agonist, such as OMDI, is used as the first anti-glaucoma medication, followed by a switch to PGs in the case of insufficient hypotensive effects by OMDI, PG-induced DUES might be avoided. However, unlike PGF2α-ags with its adverse effects, OMDI is a potential risk factor for development of cystoid macular edema, especially in patients who have undergone cataract surgery with intraocular lens implantations or who are aphakic [32,33]. In addition, the nature of 3T3-L1 cells should be different from that of human orbital fatty tissues, as suggested by a previous study [34]. Therefore, further studies directed at identifying the specific molecular mechanisms responsible for the pharmacological and pathological aspects of OMD and other EP2 agonists using human orbital fibroblasts rather than 3T3-L1 cells are now ongoing in our laboratory.

## 5. Conclusions

These collective findings indicate that the switching orders of BIM-A and EP2 agonists have a significant effect on lipid metabolism, ECM expression, and the physical stiffness of the 3T3-L1 cells.

## Figures and Tables

**Table 1:** Summary of the effects of single treatments or the switching of BIM-A and EP2 agonists on the physical properties of 3D 3T3-L1 organoids and on the adipogenesis and gene expression of 2D- and 3D-cultured 3T3-L1 cells.

		BIM-A *	OMD *	Buta *	B→O	O→B	B→Bu	Bu→B
size		↓↓↓	(−)	(−)	↓↓↓	(−)	↓↓↓	(−)
stiffness		↑↑↑	(−)	(−)	↑↑↑	↑↑↑	↑↑↑	↑↑
lipid stain	2D	↓↓	↓↓↓	↓↓↓	↓	(−)	↓↓	↑↑
	3D	↓↓	↓	↓	(−)	(−)	↓	(−)
*Pparγ*	2D	↓↓	(−)	↓	↓↓↓	↓↓↓	↓↓↓	↓↓↓
	3D	↓	↓↓	↓↓	↓↓↓	(−)	↓↓↓	↑↑↑
*Ap2*	2D	↓↓↓	(−)	(−)	↓↓↓	↓↓	↓↓↓	↓↓↓
	3D	↓	↓↓	↓↓	↓↓↓	↑↑↑	↓↓↓	↑↑↑
*Leptin*	2D	(−)	N.D	N.D	↓↓↓	(−)	(−)	(−)
	3D	(−)	N.D	N.D	(−)	(−)	(−)	↑↑↑
*Col 1*	2D	(−)	(−)	(−)	↑↑↑	↑↑↑	↑↑↑	↑↑↑
	3D	(−)	↑↑	↑↑	↑↑↑	(−)	(−)	(−)
*Col 4*	2D	(−)	(−)	(−)	↓↓	(−)	(−)	(−)
	3D	(−)	↓↓	↓↓	↑	(−)	(−)	(−)
*Col 6*	2D	(−)	(−)	(−)	(−)	(−)	(−)	(−)
	3D	(−)	↓↓	↓↓	↑	(−)	↓	(−)
*Fn*	2D	(−)	(−)	(−)	↑↑↑	↑↑↑	↑↑↑	↑↑↑
	3D	(−)	(−)	(−)	↑	(−)	(−)	(−)

BIM-A: bimatoprost acid, OMD: Omidenepag, Buta: butaprost, B→O: BIM-A to OMD, O→B: OMD to BIM-A, B→Bu: BIM-A to Buta, Bu→B: Buta to BIM-A, 2D: two-dimensional culture, 3D: three-dimensional culture, *Pparγ*: peroxisome proliferator-activated receptor γ, *Ap2*: adipocyte protein 2, *Col*: collagen, *Fn*: fibronectin, (−): non-significant change, ↑: significant increase (*p* < 0.05), ↑↑: significant increase (*p* < 0.01), ↑↑↑: significant increase (*p* < 0.005), ↓: significant decrease (*p* < 0.05), ↓↓: significant decrease (*p* < 0.01), ↓↓↓: significant decrease (*p* < 0.005), N.D.: not determined; * results are recruited from our previous studies [8,15]. Elements designated in bold with underlined results of switching are unexplained based upon the results for their mono-treatments [8,15].

## Data Availability

All data were shown in the manuscript.

## Supplementary Materials

The following are available online at https://www.mdpi.com/article/10.3390/biomedicines9121821/s1, Table S1: Sequences of qPCR primers and Taqman probes are shown.
