# Supplementary material for: Prostaglandin F2 and EP2 Agonists Exert Different Effects on 3D 3T3-L1 Spheroids during Their Culture Phase
Source: Biomedicines. 2021 Dec 2;9(12):1821. doi: 10.3390/biomedicines9121821 (PMC8698783; doi:10.3390/biomedicines9121821)
Supplement: Supplementary file 1 [file biomedicines-09-01821-s001.zip › biomedicines-1455925-supplementary.pdf]

Prostaglandin F2 and EP2 agonists exert different effects on 3D 3T3-L1 spheroids during their culture phase

Yosuke Ida, Megumi Watanabe, Araya Umetsu, Hiroshi Ohguro, Fumihito Hikage.

Departments of Ophthalmology, School of Medicine, Sapporo Medical University

All correspondence should be addressed to Fumihito Hikage

Tel# 81-11-611-2111, Fax# 81-11-613-6575, e-mail: fuhika@gmail.com

S-1 W-16, Chuo-ku, Hokkaido 060-8543 Japan

The authors declare no conflicts of interest associated with this manuscript.

Competing financial interests: Nothing to disclose.

Key words: deepening of the upper eyelid sulcus (DUES), 3T3-L1cells, PGF2α, EP2 agonists, omidenepag, 3-dimension (3D) tissue culture

Both authors (Y. I and M. W.) contributed equally to this manuscript.

Supplementary Table S1

Sequences of qPCR primers and Taqman probes are shown.

Gene	Forward Primer (5′ to 3′)	Reverse Primer (5′ to 3′)	TaqMan Probe (5′ to 3′)
Pparg	CTGCTCCACACTATGAAGACAT	TGCAGGTTCTACTTTGATCGC	/FAM/AGCTGACCC/ZEN/AATGGTTGCTGATTACA/IABkFQ/
Ap2	AAATCACCGCAGACGACAG	CCTTTCATAACACATTCCACCAC	/FAM/TGAAGAGCA/ZEN/TCATAACCCTAGATGGCG/IABkFQ/
Leptin	GTGCCTATCCAGAAAGTCCAG	AATGAAGTCCAAGCCAGTGA	/FAM/ACCGACTGC/ZEN/GTGTGTGAAATGTCA/IABkFQ/
Col1a1	CGCAAAGAGTCTACATGTCTAGG	CATTGTGTATGCAGCTGACTTC	/FAM/CCGGAGGTC/ZEN/CACAAAGCTGAACA/IABkFQ/
Col4a1	TCTGGCTGTGGAAAATGTGA	AATCCAATGACACCTTGCAAC	/FAM/TCTTTCTCC/ZEN/CTTTTGTCCCTTCACGC/IABkFQ/
Col6a1	CCAGATGAGTGTGAGATCCTG	AAGTTCTGTAGGCCAATGCTC	/FAM/ACCCATTGA/ZEN/CATCCTCTTCGTGCTG/IABkFQ/
Fn1	GAGCTATCCATTTCACCTTCAGA	TTGTTCGTAGACACTGGAGA	/FAM/CAGGAGATT/ZEN/TGTTAGGACCACGGCA/IABkFQ/
36b4	TTATAACCCTGAAGTGCTCGAC	CGCTTGTACCCATTGATGATG	/FAM/AGGCCCTGC/ZEN/ACTCTCGCTT/IABkFQ/
